# Epigenetic reprogramming of Runx3 reinforces CD8 + T-cell function and improves the clinical response to immunotherapy

**DOI:** 10.1186/s12943-023-01768-0

**Published:** 2023-05-16

**Authors:** Zongzhi Liu, Xiang Li, Yibo Gao, Jiejie Liu, Yating Feng, Yang Liu, Junyun Wang, Chunmeng Wang, Dongrui Wang, Jie He, Weidong Han, Qian Mei, Yingli Sun

**Affiliations:** 1 Central Laboratory, National Cancer Center/National Clinical Research Center for Cancer/Cancer Hospital & Shenzhen Hospital, Chinese Academic of Medical Sciences and Peking Union Medical College, Shenzhen, 518116 China; 2 Key Laboratory of Genomic and Precision Medicine, Beijing Institute of Genomics, Chinese Academy of Sciences, Beijing, 100101 China; 3 Shenzhen Key Laboratory of Synthetic Genomics, Guangdong Provincial Key Laboratory of Synthetic Genomics, CAS Key Laboratory of Quantitative Engineering Biology, Shenzhen Institute of Synthetic Biology, Shenzhen Institutes of Advanced Technology, Chinese Academy of Sciences, 518055 Shenzhen, China; 4 University of Chinese Academy of Sciences, 100049 Beijing, China; 5 Changping Laboratory, Yard 28, Science Park Road, Changping District, 102206 Beijing, China; 6 Department of Bio-therapeutic, the First Medical Center, Chinese PLA General Hospital, Beijing, China; 7 Bone Marrow Transplantation Center, The First Affiliated Hospital, School of Medicine, Zhejiang University, Hangzhou, 310058 China; 8 Liangzhu Laboratory, Zhejiang University Medical Center, Hangzhou, 310058 China

## Abstract

**Background:**

Checkpoint blockade immunotherapy, represented by PD-1 or PD-L1 antibody treatment, has been of tremendous success in clinical practice. However, the low clinical response rate and lack of biomarkers for prediction of the immune response limit the clinical application of anti-PD-1 immunotherapy. Our recent work showed that a combination of low-dose decitabine and PD-1-ab significantly improved the complete response (CR) rate of cHL patients from 32 to 71%, which indicates that there is a significant correlation between epigenetic regulation and the clinical response to immunotherapy.

**Methods:**

We recruited two groups of Hodgkin lymphoma patients who were treated with anti-PD-1 and DAC+anti-PD-1. CD8+ T cells were isolated from the patients' peripheral blood, DNA methylation was analyzed by EPIC, the expression profile was analyzed by RNA-seq, and multigroup analysis was performed with IPA and GSEA functional annotations. We explored the effect of DAC on the function of CD8+ T cells in the blood, spleen, tumor and lymph nodes using a mouse model. Furthermore, we explored the function of Tils in the tumor microenvironment. Then, we constructed Runx3-knockout mice to confirm the T-cell-specific function of Runx3 in CD8+ T cells and analyzed various subtypes of T cells and cytokines using mass cytometry (CyTOF).

**Results:**

Multiomics analysis identified that DNA methylation reprogramming of Runx3 was a crucial mediator of CD8+ T-cell function. Multiomics data showed that reversal of methylation of the Runx3 promoter promoted the infiltration of CD8+ TILs and mitigated the exhaustion of CD8+ T cells. Furthermore, experiments on tissue-specific Runx3-knockout mice showed that Runx3 deficiency reduced CD8+ T infiltration and the differentiation of effector T and memory T cells. Furthermore, Runx3 deficiency significantly decreased CCR3 and CCR5 levels. Immunotherapy experiments in Runx3 conditional knockout mice showed that DAC could not reverse the resistance of anti-PD-1 in the absence of Runx3. Moreover, both our clinical data and data from TISIDB showed that Runx3 could be a potential biomarker for immunotherapy to predict the clinical response rate.

**Conclusion:**

We demonstrate that the DNA methylation of Runx3 plays a critical role in CD8+ T-cell infiltration and differentiation during decitabine-primed PD-1-ab immunotherapy, which provides a supporting mechanism for the essential role of epiregulation in immunotherapy.

**Supplementary Information:**

The online version contains supplementary material available at 10.1186/s12943-023-01768-0.

## Highlights

1 We demonstrated that Runx3 is the key mediator of low-dose decitabine (DAC)-primed-anti-PD-1 immunotherapy and that epigenetic reprogramming of Runx3 significantly improves the clinical response to anti-PD-1 immunotherapy.

2 Both in vitro and in vivo conditional knockout data of Runx3 showed that Runx3 plays an essential role in CD8+ cell differentiation and infiltration. Knockout of Runx3 reduced effector T and memory T numbers and CCR3 and CCR5 levels. Runx3 plays a critical role for significant improvement of clinical response of DAC primed anti-PD-1 treatment.

3 Our work shows that the Runx3 level can be used to predict the immune response to immune checkpoint blockade therapies.

**Supplementary Information:**

The online version contains supplementary material available at 10.1186/s12943-023-01768-0.

## 1 Introduction

Cancers are highly complex diseases that are characterized by not only the overgrowth of malignant cells but also an altered immune response. The inhibition and reprogramming of the immune system play critical roles in tumor initiation and progression. Immunotherapy aims to reactivate antitumor immune cells. Tumor immunotherapies, representative strategies of immune checkpoint blockade and adoptive cell transfer, have seen tremendous success in clinical practice, with the capability to induce long-term regression of some tumors that are refractory to all other treatments. Among them, PD-1-ab treatment has been the most comprehensively applied clinical immunotherapy for various types of cancers [1–8]. Inhibition of the function of PD-1 with PD-1 antibodies can activate T cells to treat cancer. Thus far, many PD-1 or PD-L1 inhibitors have been approved. The emergence of PD-1 and PD-L1 antibodies has greatly changed the status of cancer treatment [8–13].

Anti-PD1/PDL1 immunotherapy has become one of the most popular treatments among various lines of treatment for tumor patients, but it has also produced a large number of immune resistance patients. How to arrange follow-up treatment for such patients and whether immunotherapy can be extended or resensitization can be accomplished are major practical problems for clinical treatment. Previous work has shown that 25% of patients with solid tumors and 40–60% of patients with certain lymphomas respond to current anti-PD therapy [14–21]. The objective response rate (ORR), however, is quite variable across the various types of cancer [14–21]. For instance, the ORR to pembrolizumab is 56% in Merkel cell carcinoma and 45% in advanced-stage melanoma but approximately 20% in advanced-stage NSCLC and just 16% in gastroesophageal junction carcinoma. Therefore, it is important to precisely identify patients who will or will not respond before therapy is conducted. The clinical response rate of PD-1 antibody therapy in relapsed and refractory Hodgkin lymphoma is only 15-31% [17, 18]. How to improve the clinical response rate of PD-1 antibodies in refractory Hodgkin lymphoma is an important issue. It is urgent to elucidate the mechanism of immunotherapy to change the clinical response and provide biomarkers.

Recent work has shown that epigenetic regulation of T-cell function plays a critical role in T-cell activation and T-cell exhaustion [22–39]. It has been reported that de novo DNA methylation promotes T-cell exhaustion, whereas methylation inhibition by decitabine (DAC) enhances PD-1-blockade-mediated-T-cell-rejuvenation [30]. However, the specific target of epigenetic regulation of T-cell function remains unclear. It is also essential to link clinical observations to molecular studies. The above work was performed in mice. Considering the differences in immune systems in mice and humans, we initiated our work with clinical samples. Decitabine is currently the strongest specific inhibitor of DNA methylation [40–42] Decitabine (DAC), also known as 5-aza-2'-deoxycytidine, can integrate into DNA and bind to DNA methyltransferase, inhibiting its function [41–46]. Our previous work showed that a combination of decitabine and PD-1-ab enhanced the antitumor efficacy of PD-1 inhibitors and significantly increased the complete remission rate of relapsed and refractory Hodgkin lymphoma from 32 to 71% [1]. Seventy percent of patients who failed PD-1 inhibitor monotherapy responded again, and 28% of patients achieved complete remission [1]. Our later work confirmed that DAC also promoted CAR-T immunity, but the mechanism remains unclear [2].

Through multiomics screening, we found that Runx3 (Runt-related transcription factor 3) promoter hypermethylation inhibited the expression of Runx3 and correlated with anti-PD-1 resistance. As a specific inhibitor of DNA methylation, decitabine could reverse the hypermethylation state of Runx3 and increase the expression of Runx3. The epigenetic reprogramming of Runx3 thereby augmented the function of T cells and promoted infiltration, which led to a significant change in clinical outcome.

Transcriptional regulation of Runx3 is dominantly controlled by promoter methylation. The Runx3 gene is a recently discovered tumor suppressor gene [46–48]. It belongs to the Runt domain (RD) transcription factor family and is involved in the regulation of cell growth and apoptosis [48]. The transcription of the Runx3 gene is mainly controlled by the promoter P2. P2 is located before exon 2, with a GC content of approximately 64% and a CpG island around it [47, 48]. Studies have confirmed that there is low expression of Runx3 in many tumors and that the main reason for the low expression of Runx3 is hypermethylation of the CpG island in the Runx3 promoter region [46–48].

In recent years, studies have found that Runx3 is also involved in the occurrence and development of various immune cells and plays important roles in their occurrence, development, and functional activation [49–54]. Previous data have revealed that Runx3 is one of the key regulators of the fate choice between T_eff_ and T_ex_ after initial activation. Runx3 is also an important regulator of T-cell residency [49]_._ However, although Runx3 expression changes have been observed in many single-cell and CRISPR screening studies on T-cell function, there is no clear evidence linking the clinical role of Runx3 to T-cell infiltration and T-cell exhaustion.

CCRs (chemokine receptors) are cytokine receptors found on the surfaces of certain cells that interact with a type of cytokine called a chemokine. There have been 19 distinct chemokine receptors described in mammals [55]. A variety of chemokine/chemokine receptor strategies have been used in preclinical studies of immunotherapeutic T cells to promote the targeting of CAR-T cells to tumors, including for the use of CXCR3, CXCR2, CCR5, CCR2, and CCR3 axes [55]. Our in vivo work showed that in addition to regulating the differentiation of effector T and memory T cells, knockout of Runx3 blocked T-cell infiltration and downregulated CCR3 and CCR5. Consequently, low-dose decitabine could not restore the function of T cells in Runx3-deficient mice, and decitabine could not release anti-PD-1 resistance in knockout mice.

The overall impressive clinical effect of anti-PD-1 has led to several approvals of related treatments. However, not all patients can benefit from anti-PD-1 treatment, making it critical to identify biomarkers for efficacy prediction. Biomarkers are critical for screening and classifying patients, accurately identifying patients with a drug response, and enabling patients to receive the best treatment as soon as possible. Our work shows that the methylation level of Runx3 and the expression level of Runx3 can predict the immune response to immune checkpoint blockade therapies.

Overall, we have demonstrated that methylation reprogramming of Runx3 by decitabine plays a key role in improving the therapy response with a combination of low-dose decitabine and anti-PD-1 and that the expression level of Runx3 is a potential biomarker for the anti-PD-1 therapy response.

## 2 Materials and Methods

### 2.1 Preparation of patient samples and ethics approval

Peripheral blood samples were collected, and CD8+ T cells were sorted before and after treatment. All samples were collected from Chinese PLA General Hospital (Beijing, China) with the informed consent of the patients, and the experiments were approved by the Ethics Committee of the Chinese PLA General Hospital. (ClinicalTrials.gov identifier: NCT02961101 and NCT03250962). Blood samples were collected for peripheral T-cell assessment before treatment in each of the first two cycles (Cnd0, n indicates 1 to 2) and after decitabine administration but before anti-PD-1 infusion in the first two cycles of the combination group. In total, 10 samples were collected for high-throughput molecular analyses. Detailed information about the 10 patients used for EPIC and RNA-seq is provided in Supplementary Table 1.

### 2.2 Construction of the mouse model

All animal work was undertaken in accordance with the National Institutes of Health's Guide for the Care and Use of Laboratory Animals, with the approval of the Scientific Investigation Board of Chinese PLA General Hospital. Wild-type C57BL/6J mice of both sexes were purchased from Biocytogen Pharmaceuticals (Beijing) Co., Ltd. Runx3 flox mice were generated by a CRISPR/Cas9-based approach. Briefly, two sgRNAs were designed by the CRISPR design tool (http://www.sanger.ac.uk/) to target either the upstream or downstream region of the transcript NM_019732.2 exon 4 of mouse Runx3 and then screened for on-target activity using a Universal CRISPR Activity Assay (UCATM, Biocytogen Pharmaceuticals (Beijing) Co., Ltd). The T7 promoter sequence was added to the Cas9 or sgRNA template by PCR amplification in vitro. The donor vector containing exon 4 flanked by 2 loxP sites and 2 homology arms (left, 1407 bp; right, 1476 bp) was used as a template to repair the DSBs generated by Cas9/sgRNA. Each loxP site was located 3 nt upstream of the PAM site (cleavage site of Cas9 nuclease). Cas9 mRNA, sgRNAs and donor vectors were coinjected into the cytoplasm of one-cell stage fertilized C57BL/6N mouse embryos. Through subsequent hybridization and genotype identification with Lck-cre mice, Runx3^fl/fl;Lck-Cre^ mice were finally constructed.

### 2.3 Preparation of DNA and RNA samples

Each peripheral blood sample was obtained following the protocol of the clinical trial. Peripheral blood mononuclear cells (PBMCs) were isolated from 10 ml of whole blood by centrifugation. CD8+ T cells were sorted with a Human CD8+ T-Cell Isolation Kit (Miltenyi). Genomic DNA was extracted from whole blood using a QIAamp DNA Mini Kit (Qiagen) according to the manufacturer’s instructions. Total RNA was extracted with TRIzol Reagent (Invitrogen). DNA and RNA were quality assessed using an Agilent Bio Analyzer 2100, and quantification was performed using a NanoDrop ND-1000 spectrophotometer.

### 2.4 EPIC BeadChip methylation

One microgram of DNA was bisulfite-converted using an EZ DNA Methylation-Gold Kit (ZYMO), and the DNA was whole-genome amplified, enzymatically fragmented, purified, and applied to an Illumina Infinium Methylation EPIC BeadChip Array according to the Illumina methylation protocol. DNA methylation IDAT files were processed in R using the minfi package. Probes with fewer than three beads for either the methylated or unmethylated channel or with a detection *P *value ≥ 0.01 were removed. Probes with SNPs or their single base extension, X chromosome, or Y chromosome at the CpG site were excluded. After quality control filtering, 829,120 CpGs in 25 samples remained for later analysis. DNA methylation files were processed and normalized by R software packages using the ChAMP package. For each of the samples, CpG sites with a detection *P* value less than 0.05 were excluded from the analysis. In addition, probes with SNPs or their single base extension, X chromosome and Y chromosome at the CpG site were excluded. The standard DMSs were δ beta| > 0.1 and *p* < 0.05 (Wilcoxon test).

### 2.5 RNA sequencing

The sequencing quality of RNA-Seq libraries was assessed by FastQC v0.10.1 (http://www.bioinformatics.babraham.ac.uk/projects/fastqc/). RNA-seq libraries were mapped to the human genome using TopHat (v2.1.0), and the mapped reads were then processed by HISAT and StringTie to estimate the expression levels of all genes and identify differentially expressed genes. The expression level of a gene is expressed as a gene-level fragments per kilobase of transcripts per million mapped reads (FPKM) value. Since there were only 3 samples in each group, upregulated or downregulated genes in the patient’s CD8+ T cells were identified by requiring ≥ 2-fold expression changes to explore the overall differences.

### 2.6 Pathway analysis

The biological relevance of gene groups comprising modules identified by differentially methylated genes and differentially expressed RNA genes was further investigated using the Ingenuity Pathways Analysis platform. The bubble chart was drawn by the R package.

### 2.7 Real-time PCR

CD8+ T cells from 48 immunotherapy-responsive and nonresponsive patients were isolated, and total RNA was extracted. The qRT‒PCR assay for gene expression was performed with SYBR Green Real-time PCR Master Mix (ToYoBo). β-Actin was used as an internal control within the 2^-ΔΔCt^ cycle threshold method. Independent-sample t tests were used to compare responders (including patients with a complete response (CR) and a partial response (PR)) and nonresponders (patients with stable disease (SD) and progressive disease (PD)). The primers used are shown in Table 2.

### 2.8 MSRE-qPCR Conditions

MSRE-qPCR was carried out as previously described [56]. The primers were as follows: F: CTGAACCTTTTAAGAGAGCC R CAAATGGAATTTACCACCAC. The methylation level of the Runx3 promoter was determined using OneStep qMethyl Kit from Zymo Research. Analyses were performed in duplicate for each experiment. The final reaction volume of 20 µL and contained premix with SYTO 9 dye, 10 pmol/µL of each primer, and 5 µL of DNA template. Reactions were carried out in the presence (test reaction) or absence (reference reaction) of MSRE (AccII, HpaII, and HpyCH4IV), as per the manufacturer’s guidelines. The cycling conditions were as follows: MSRE digestion (37 °C 2h), initial denaturation (95 °C, 10 min), 40 cycles of three-step amplification (denaturation: 95 °C, 30 s; annealing: 54 °C, 60 s; extension: 72 °C, 60 s), and final extension (72 °C, 7 min). In addition, the amplified products were melted in a temperature gradient to a maximum of 95 °C.

### 2.9 Database analysis

The potential of the expression of Runx3 in cancer samples to predict the response to anti-PD-1/L1 antibody treatment was evaluated with TIDE analysis. The transcriptomes of Runx3 were analyzed in the TISIDB database. The Kaplan‒Meier method was used to analyze the association between the expression of Runx3 and overall survival (OS) as well as progression-free survival (PFS) in breast cancer, ovarian cancer, colon cancer, lung cancer, diffuse large B-cell lymphoma (DLBC) and chronic lymphocytic leukemia (CLL). The relations between the abundance of tumor-infiltrating lymphocytes (TILs) and the expression of Runx3 were analyzed in 8 cancer types in the TISIDB dataset.

### 2.10 In vivo experiments

MC38 cell lines were obtained from the National Infrastructure of Cell Line Resource of China. A total of 5 × 10^5^ MC38 cells were resuspended in Matrigel to a final volume of 100 mL and then injected subcutaneously into the flanks of 5- to 6-week-old wild-type C57BL/6 or Runx3^fl/fl^ and Runx3^fl/fl;Lck-Cre^ mice. The mice were randomized into four groups (six mice/group) when the tumors reached an approximate average volume of 100-150 mm^3^. Decitabine was administered intraperitoneally (i.p.) for 5 consecutive days at 0.2 mg/kg/day and was followed by anti-PD-1 antibody (200 μg) or PBS every 3 days 3 times. For anti-PD-1 monotherapy, mice were treated with 200 μg of anti-PD-1 antibody every 3 days 3 times after grouping. The tumor volumes were calculated using the following formula: volume = length×width^2^/2.

### 2.11 Flow cytometry analysis (FACS)

Single-cell suspensions were prepared from mouse peripheral blood, spleen, lymph nodes and tumor. T-cell cytokine expression was determined by intracellular staining. Cells were stained with specific antibodies listed in Table 3. Doublets and debris of dead cells were excluded before various gating strategies were applied. Immunophenotyping was performed using BD FACSCalibur, and data were analyzed using Kaluza Analysis2.0.

### 2.12 Mass Cytometry (CyTOF)

After fresh tissue was obtained, it was immediately placed and completely immersed in a 2 ml cryopreservation tube. Fresh tissue was refrigerated (2~8 ℃) for inspection after collection. The tissue preservation solution was discarded, 1~2 ml of 1640 basic medium was added, and the tissue was washed twice. The tissue was cut into 1 mm^3^ fragments with ophthalmic scissors, Miltenyi tumor dissociation reagent was added, and the mixture was supplemented with 1640 basic medium to 5 ml. The digestion conditions were 37 ℃, 145 rpm, and 1 h. A 70 μM sieve was used to filter the digestive solution into the collection pipe. Regarding the red blood cells in the precipitate, 1 ml of ACK was added (terminated within 1 min), and the cells were centrifuged for 5 min at 400×g. FACS buffer was used to resuspend the cells, and 10 μL was used to count (2 times) living cells and dead cells. The collection tube was centrifuged for 10 min at 400×g to obtain cell precipitates. Then, the cells were stained according to the antibody requirements, and all mass cytometry files were normalized and manually gated in FlowJo (version X 10.0.7r2) or Cytobank (Santa Clara, CA). t-Distributed stochastic neighbor embedding (t-SNE) dimension reduction was performed using the R package. The statistical analysis selected in this project included a two-sided t test or Wilcoxon rank sum test. When *p* < 0.05, there was a significant difference in the average percentage of specific cell subsets between the two groups.

## Results

### Multiomics data analysis from a clinical cohort showed that Runx3 is the key mediator of improved clinical response with low-dose DAC-primed anti-PD-1 immunotherapy

**Fig. 1:**
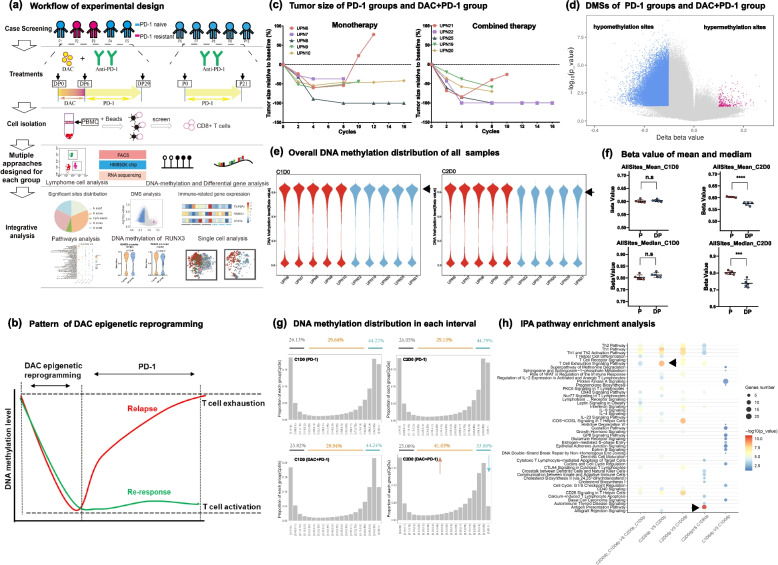
DAC can trigger large-scale apparent reprogramming of CD8+ T cells. **a** Workflow of the experimental design including clinical sample collection and sequencing. **b** Schematic chart showing that DNA methylation reprogramming is correlated with clinical response and relapse. **c** Correlation analysis of tumor size and the anti-PD-1/DAC treatment cycles of patients. The patients were treated as indicated. Tumor size increase>50% were considered to indicate progression. **d** Analysis of genome-wide methylation variations in CD8 + T cells between the two indicated groups. The methylation was screened according to a |Diff beta value >0.1 and *P*< 0.05. Blue represents hypomethylation sites, and red represents hypermethylation sites. **e** Violin diagram showing the genome-wide methylation distribution of each patient. Red represents the monotherapy group, and blue represents the combined therapy group. The left panel is the baseline period of C1D0, and the right panel is the end of C2D0 treatment. **f** Statistical analysis of genome-wide DNA methylation levels. Upper panel: mean value of DNA methylation in the C1D0 and C2D0 periods; lower panel: median value of DNA methylation in the C1D0 and C2D0 periods (two-tailed unpaired t tests. n.s.: not significant, ***P < 0.001, ****P < 0.0001) **g** Distribution analysis of DNA methylation levels after anti-PD-1 or anti-PD-1/DAC. The DNA methylation value was divided into 20 sections from 0-1. A value < 0.15 was taken as the low methylation level, and a value > 0.85 was taken as the high methylation level. Upper panel: treated with anti-PD-1. Lower panel: treated with anti-PD1/DAC (left panel: before treatment; right panel: after treatment). **h** IPA pathway enrichment analysis. The input data are DMSs in each period. The size of the circle shows the number of enriched genes in each pathway, and the color depth represents the degree of enrichment

We recruited two groups of patients treated with anti-PD-1 vs. anti-PD-1/DAC (DP). DNA methylation EPIC and RNA-seq were performed as shown in the workflow (Fig. 1a). To explore the DNA methylation reprogramming profile of CD8+ T cells in patients, we obtained the DNA methylation profile of CD8 + T cells from anti-PD-1-treated patients versus patients treated with primed DAC (Fig. 1a). Compared to anti-PD-1-treated patients, large-scale demethylation was detected in DAC-primed anti-PD-1-treated patients, and clinical response was evaluated in correlation with treatment (Fig. 1a-c). The number of hypomethylation sites reached 113972, and these genome-wide demethylation changes were mainly distributed on N-shelf and S-shelf (Fig. S1e).

Furthermore, we identified 295 hypomethylated DMSs, while the number of hypermethylated DMSs was 951. Moreover, we found a significantly increased number of hypomethylation sites in the DP combined treatment group (Fig. 1d, Fig. S1b), which suggests that large-scale demethylation of CD8+ T cells occurred after DP treatment.

To further explore the dynamic profile of DNA methylation reprogramming and its correlation with clinical outcome, we compared DMSs at the C1D6 (5 days after DAC treatment) stage vs. the C1D0 (baseline before treatment) stage and the C2D0 (after one cycle of anti-PD-1 treatment) stage vs. C1D6 (5 days after DAC treatment) stage. The results showed that large-scale demethylation occurred after DAC treatment (Fig. S1c). In contrast, dynamic DNA methylation reprogramming was not observed in the PD-1 blockade monotherapy group (Fig. S1d). Furthermore, to confirm DNA methylation reprogramming in individual patients, we quantitatively analyzed the genome-wide methylation levels and differential methylation levels of each patient (Fig. 1e). The results showed that all CD8+ T cells of all patients in the DP group had significant demethylation profiles during C2D0 (after one cycle of anti-PD-1 treatment), which implied that the demethylation caused by DAC showed no individual differences. Furthermore, the methylation level was analyzed with statistical analysis, and significant difference was observed upon treatment with anti-PD-1 vs. anti-PD-1/DAC (Fig. 1f). Analysis of the distribution of DNA methylation showed that significant DNA demethylation occurred in high methylation sites when anti-PD-1/DAC treatment was conducted (Fig. 1g).

We then performed Ingenuity Pathway Analysis on DMSs, which showed that DMSs were enriched mainly in T-cell exhaustion- and T-cell activation-related pathways, including the T-cell exhaustion signaling pathway, Th1 and Th2 activation pathways, and in the role of NFAT in the regulation of the immune response (Fig. 1h). Furthermore, we also analyzed the C2D0 (after one cycle of anti-PD-1 treatment) vs. C1D0 (baseline before treatment) status in CD8+ T cells in the DP group. The DMSs were also mainly enriched in the T-cell exhaustion signaling pathway, CTLA4 signaling in cytotoxic T lymphocytes, interferon signaling, and interferon signaling (Fig. 1h). The above results showed that DAC treatment may result in the reversal of T-cell exhaustion and augment T-cell activation and infiltration.

**Fig. 2:**
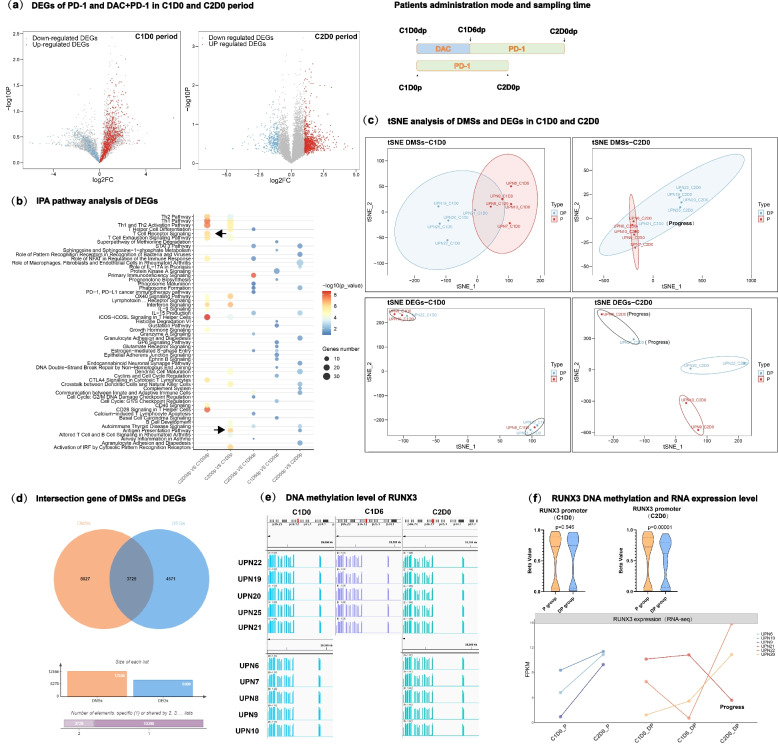
Expression profile and integrated multiomics analysis in CD8 + T cells identified important signaling pathways in response to DAC treatment. **a** The expression fold changes of DEGs increased at different stages. Left panel: Expression of differentially expressed genes in the C1D0 period in anti-PD-1-vs. anti-PD-1/DAC-treated patients. Right panel: Expression of differentially expressed genes in the C2D0 period in anti-PD1-vs. anti-PD-1/DAC-treated patients. Red indicates up-regulated genes, and blue indicates down-regulated genes. **b** IPA pathway enrichment analysis. The input data are the DEGs in each period. The size of the circle shows the number of enriched genes in each pathway, and the depth of the color represents the *P*-value of enrichment. **c** Workflow of the experimental design and tSNE analysis of DMSs and DEGs in the C1D0 and C2D0 periods. Blue represents the combined therapy group with DAC and anti-PD-1, and red represents the monotherapy group with anti-PD-1. Upper panel: tSNE analysis of DMSs. Lower panel: tSNE analysis of DEGs. **d** Intersecting gene analysis of DMSs and DEGs. Orange represent DMSs, and blue represents DEGs. **e** IGV showed Runx3 methylation levels in different patients at different stages. **f** Correlation of gene expression and the promoter methylation level of Runx3. Upper panel: Violin diagram showing the statistical analysis of the difference in methylation levels in the Runx3 promoter region. The figure shows the median, upper quartile and lower quartile. Two-tailed unpaired t tests. Lower panel: The expression levels of Runx3 in different periods were analyzed by a line diagram. The x-axis represents the period, and the y-axis represents the FPKM value

To investigate whether DNA methylation reprogramming correlates with gene expression, we performed RNA-Seq and analyzed the expression profiles of CD8 + T cells. The results showed that there were a large number of differentially expressed genes (DEGs) in the two groups of CD8+ T cells. As shown in the volcano map, we found that the expression fold changes of DEGs increased significantly in the C2D0 (after one cycle of anti-PD-1 treatment) period (Fig. 2a). By analyzing the differences in the DP group and comparing C2D0 (after one cycle of anti-PD-1 treatment) versus C1D0 (baseline before treatment), we found downregulated genes such as CCR2, TNFSF14, and TNFSF4 and upregulated genes such as CCL3, TIGIT IFNG and CD69 and cytokines such as CCR3 and CCR5 were upregulated significantly under DAC treatment, and TOX was downregulated under DAC treatment. These data indicate that exhausted T cells can be reversed by DAC and that infiltration-related cytokines are upregulated by DAC but not anti-PD-1. We further performed IPA pathway enrichment analysis (Fig. 2b) and GSEA (Figure S2b) to explore the possible biological significance of these findings. The results showed that these differences were enriched in the T-cell exhaustion pathway, Th1 and Th2 activation pathway and T-cell receptor pathway, which was highly consistent with the enrichment results of DMSs in DNA methylation profiling. Through tSNE analysis, we found that the CD8+ T cells consisted of two distinct groups after anti-PD-1 treatment (Fig. 2c).

Through multiomics joint analysis of DEGs and DMSs, we found 3729 genes that intersected in terms of DNA methylation and expression profile (Fig. 2d). Through pathway analysis, we found that the pathways were mainly enriched in the regulation of T-cell activation, cytokine signaling in the immune system, immune system development, and regulation of the leukocyte cell‒cell adhesion pathway, which suggests that the combination of DP may play a central role in the development, activation and response to cytokines of the immune system.

Due to the consistency between DMSs and DEGs, we conducted a joint analysis of the two omics datasets. We found that Runx3 was the most significantly regulated gene. After treatment with DAC, all CR patients maintained stable demethylation levels on the Runx3 promoter and high expression levels of Runx3 (Fig. 2e). The correlation of DNA methylation and expression was confirmed (Fig. 2f). DNA demethylation was also observed in UPN21; however, the state of demethylation could not be well maintained as in this patient, and the expression level of Runx3 was downregulated back to baseline levels. Then, we found that this patient had disease relapse after 6 months, which highlights the importance of DNA methylation status for clinical outcome. The above case of recurrence showed a clear correlation between DNA methylation reprogramming and clinical outcomes, and epigenetic reprogramming of the Runx3 promoter plays a key role in regulating Runx3 expression during DAC-primed anti-PD-1 treatment.

### In vivo work in mice demonstrated that DAC treatment promoted T-cell infiltration and downregulated T-cell exhaustion

**Fig. 3:**
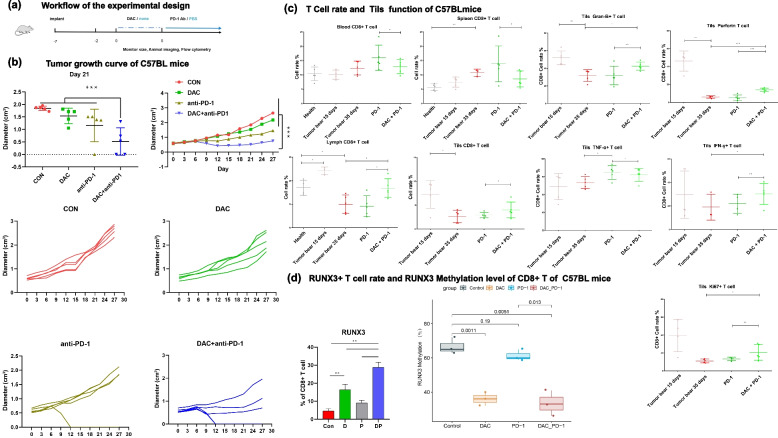
DAC downregulated T-cell exhaustion and upregulated T-cell infiltration by demethylating Runx3 and promoting Runx3 expression. **a** Workflow of the experimental design and analysis of the tumor growth curve using the MC38 mouse model treated with DAC, anti-PD-1 or DAC/anti-PD-1(*n*= ). b Tumor growth curve of mice treated with DAC, anti-PD-1 or anti-PD-1/DAC. Upperpanel: average tumor growth curves;(two-tailed unpaired t tests, *P <0.05, **P<0.01, ***P<0.001) **c** The proportions of GranB+, perforin+, TNF-α+, IFN-γ+, Ki67+ and CD8+ T cells were analyzed by flow cytometry. Samples were taken from the blood, spleen, tumor, or lymphocytes of MC38 mice treated with DAC, anti-PD1 or anti-PD-1/DAC as indicated. (*n*=5, two-tailed unpaired t tests, *P <0.05,**P <0.01***P < 0.001). **d** Leftpanel: The proportion of Runx3+CD8+ T cells in each group was analyzed by flow cytometry (*n*=5, two-tailed unpaired t tests, **P<0.01). Right panel: DNA methylation level change on Runx3 promoter in T cells treated with DAC, anti-PD-1 or antiPD-1/DAC. Y axis: Mehylation level of Runx3 (%); X axis: sampes of mice treated with DAC, anti-PD-1 or DAC/anti-PD-1. Triplicate samples were applied for each experiment and the median was shown as horizontal line within the box plots. (*p*<0.05)

To further investigate the underlying mechanism of the “epigenetic sensitization” role of DAC immunotherapy, we aimed to reproduce the clinical observation in mice and establish an in vivo mouse model. C57BL/6 mice were implanted with MC38 cells and treated with either DAC, anti-PD-1 or anti-PD-1/DAC at the indicated times, simulating the clinical situation of patients (Fig. 3a).

As shown in Fig. 3b and c in MC38 tumor-transplanted mice, we found that DAC combined with anti-PD-1 significantly inhibited tumor growth, promoting the infiltration of TILs. In the MC38 model, anti-PD-1 treatment showed insignificant inhibition of tumor growth, but the combination of DAC and anti-PD-1 significantly inhibited the growth of tumors. We further analyzed the function of CD8+ T cells with flow cytometry. As shown in Fig. 3c, we found that the proportion of proliferative T cells increased significantly (ki67+CD8+ T). The proportions of killing (GranB+CD8+T, proferin+CD8+ T) and secretory cells (IFN-γ+CD8+ T) cells were also significantly improved.

Our results also showed that the DNA methylation status of Runx3 decreased significantly in DAC- and DAC/anti-PD-1-treated mouse CD8+ T cells but not in WT and anti-PD-1-treated mouse CD8+ T cells (Fig. 3d).

To explore the immune status of the peripheral immune system and tumor microenvironment, we examined the number and function of T cells in lymph nodes, spleens, peripheral blood and tumor. The results showed that the numbers of CD3+ T cells in the blood, spleen, and lymph gland were decreased in the combination therapy group. The numbers of CD8+ T cells were decreased in the spleen and blood and increased in the lymph gland in combination therapy. This indicated that the proliferation, killing and secretion of interferon by tumor-infiltrating T cells were all significantly enhanced, indicating the overall recovery of T-cell function in the tumor microenvironment (Figure 3c). Flow cytometry showed that the proportion of Runx3+CD8+ T cells increased more when cells were treated with DAC than when cells were with anti-PD-1 and peaked when cells were treated with both DAC and anti-PD-1 (Figure 3D).

### Conditional knockout of Runx3 proved that Runx3 is indispensable for DAC to play the role of “epigenetic sensitizer” for anti-PD-1 resistance

To rule out the antitumor role of Runx3 in cancer cells, we constructed conditional knockout mice to prove the specific function of Runx3 in T cells and immunotherapy.

**Fig. 4:**
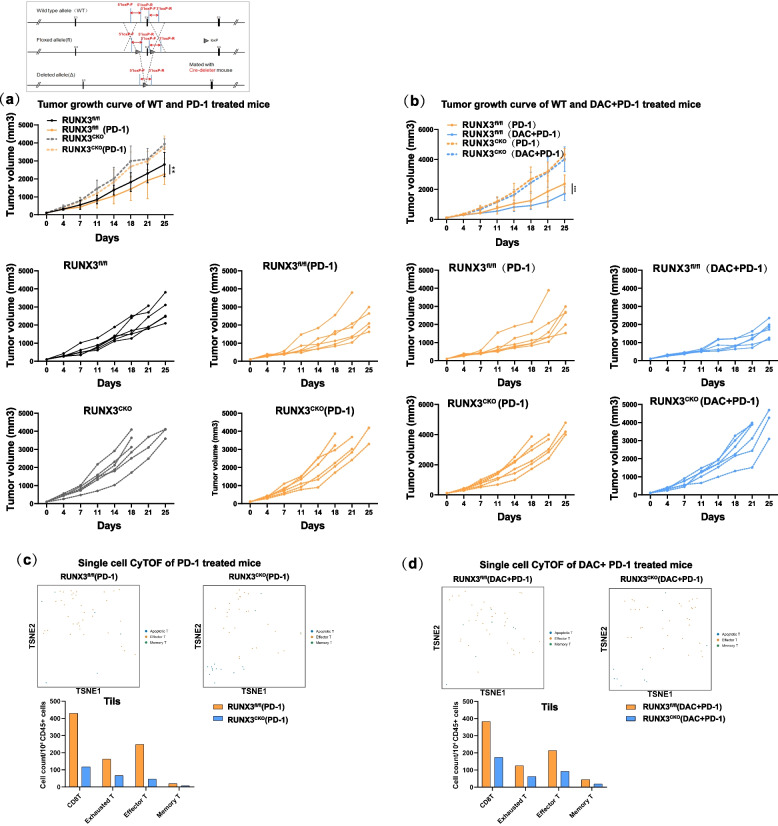
The epigenetic sensitization effect of immunotherapy was eliminated in Runx3^fl/fl;Lck-Cre^ mice. **a** Workflow of the construction of Runx3 conditional knockout mice and analysis of the tumor growth curve in Runx3^fl/fl^ and Runx3^fl/fl;Lck-Cre^ mice treated with anti-PD-1(*n*=5, two-tailed unpaired t tests, **P< 0.01). Upper panel: average tumor growth curves; lower panel: individual tumor growth curves. **b** Analysis of the tumor growth curve in Runx3^fl/fl^ and Runx3^fl/fl;Lck-Cre^ mice treated with anti-PD-1or DAC+ anti-PD-1(*n*=5,two-tailed unpaired t tests, **P<0.01). Upper panel: average tumor growth curves; lower panel: individual tumor growth curves. **c** tSNE analysis of immune cell subsets in the CD8+Tils of Runx3^fl/fl^ and Runx3^fl/fl;Lck-Cre^ mice treated with anti-PD-1. Upper panel: tSNE data showing the overall distribution of each subgroup. Lower panel: Histogram showing the absolute numbers of cells of various subtypes (cell number/10^4^ CD45+ cells). **d** tSNE analysis of immune cell subsets in the CD8+ Tils of Runx3^fl/fl^ and Runx3^fl/fl;Lck-Cre^ mice treated with DAC+anti- PD-1. Upper panel: tSNE data showing the overall distribution of each subgroup. Lower panel: Histogram showing the absolute numbers of cells of various subtypes (cell number/10^4^ CD45+ cells)

Runx3 flox mice were generated by a CRISPR/Cas9-based approach. Briefly, two sgRNAs were designed with the CRISPR design tool (http://www.sanger.ac.uk/) to target either the upstream or downstream region of the transcript NM_019732.2 exon 4 of mouse Runx3. Through subsequent hybridization and genotype identification with Lck-cre mice, Runx3^fl/fl;Lck-Cre^ mice were finally constructed. The Runx3^fl/fl^ mice showed no difference in weight or development. No autoimmune disease was observed. Then, both Runx3^fl/fl^ and Runx3^fl/fl;Lck-Cre^ mice were divided into three groups: the control, anti-PD-1-treated, and anti-PD-1/DAC-treated group (Fig. 4a).

The antitumor immunity was decreased in Runx3^fl/fl^^;Lck-Cre^ mice (Fig. 4a, b). There were significant differences between the anti-PD-1 group and DAC/anti-PD-1 group in control Runx3^fl/fl^ mice (Fig. 4c, d). In addition, there was no significant difference in tumor growth curve between mice in the DAC-primed anti-PD-1 and anti-PD-1 group in Runx3^fl/fl;Lck-Cre^ mice. No significant differences in tumor volume and mass were observed, which indicated that the effect of DAC was eliminated after conditional knockout of the Runx3 gene.

To further investigate the role of Runx3 in CD8 + T cells, we applied single-cell flow mass spectrometry analysis (CyTOF) and found that the proportion of CD8+ tumor infiltrating lymphocytes (TILs), changed significantly. Comprehensive analysis suggested that Runx3 increased the proportion of T^eff^ and T^RM^ cells and interfered with the balance of immune cell subtypes (Fig. 4c, d).

### Runx3 plays a critical role in T-cell infiltration and effector and memory T-cell differentiation and functions to attenuate T-cell exhaustion

Tumor immunotherapy consists of multiple steps of the T-cell functional response. First, T cells differentiate into effector T cells and then memory T cells to exert antitumor functions. Second, it is essential for T cells to infiltrate into tumors to kill tumor cells. A lack of immune cells in the tumor microenvironment is an important reason for the low response. Third, T cells are often in a state of exhaustion or dysfunction [17–21], and the exhaustion of T cells affects the PD-1 antibody response rate as well [28]. To elucidate the specific role of Runx3, we performed mass cytometry (CyTOF) to compare the T-cell function of conditional knockout mice and control mice. We employed 42 markers to cover cytokines, T-cell exhaustion markers, T-cell proliferation and T-cell killing ability. Since we observed a significant increase in CCR after DAC treatment and we found that DAC promoted T-cell infiltration in our mouse model, several CCRs were included among these 42 markers.

**Fig. 5:**
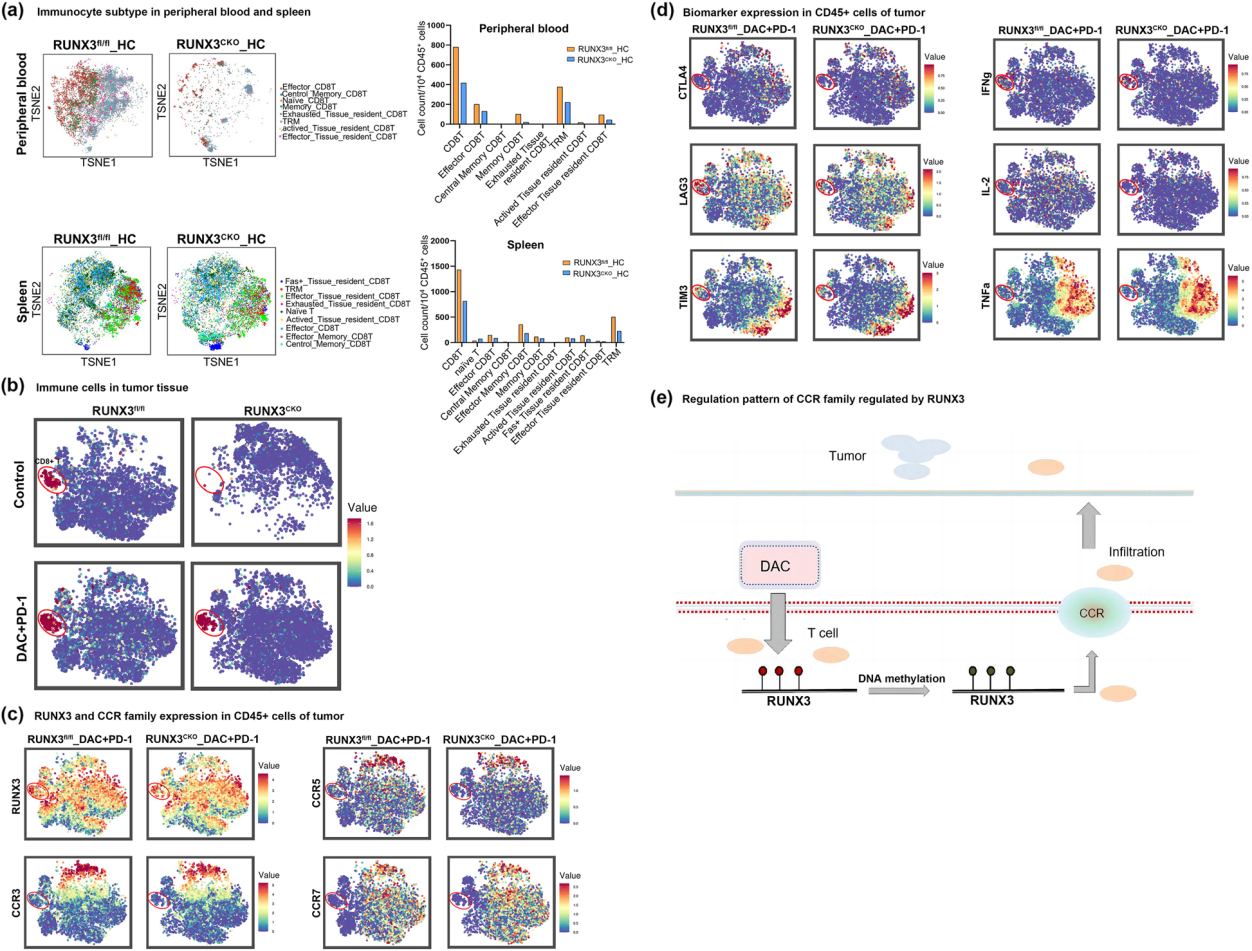
Runx3 deletion hampers CCRs expression and tumor infiltration of CD8+ T cells. **a** Determination of immune cell subsets in the peripheral blood and spleen of Runx3^fl/fl^ and Runx3^fl/fl;Lck-Cre^ (Runx3^CKO^)mice by tSNE analysis after mass cytometry. Left panel: tSNE data showing the overall distribution of each subgroup. Right panel: The histogram shows the absolute numbers of cells of various subtypes (cell number/10^4^ CD45+ cells). **b** CD8+ T cells distribution in the tumor tissue of Runx3^fl/fl^ and Runx3^CKO^ mice by tSNE analysis. **c** tSNE plots showing Runx3 and CCRs expression of T cells after anti-PD-1/DAC treatment. The plots represented CD45+ immune cells in mice tumors, and the circle indicated CD8+ T cell population. **d** tSNE plots showing expression of marker genes of T cells after anti-PD-1/DAC treatment. The plots represented CD45+ immune cells in mice tumors, and the circle indicated CD8+ T cell population. **e** Schematic illustration showing that demethylation of Runx3 by DAC promoted CCRs expression and T-cell infiltration

First, we observed significant downregulation of CD8+ T cells and effector T cells in peripheral blood, spleen, tumor tissue of mice with Runx3 deficiency (Fig. 5a, b). Furthermore, we found that Runx3 deficiency significantly downregulated CCR3 and CCR5, consequently impairing T-cell infiltration (Fig. 5c). Deficiency of Runx3 also impaired T-cell function by affecting T-cell differentiation and T-cell exhaustion (Fig. 5d). We observed increased levels of Lag3, Tim3 and CTAL4 but decreased levels of IFNγ, TNFα and IL-2. Interestingly, we did not find increased levels of PD-1 in Runx3-deficient mice. Previous work has shown that PD-1 is expressed only after T cells are activated, while Runx3 deficiency significantly downregulates effector T cells and memory T cells, which in turn might balance the expression level of PD-1 to increase exhausted T cells.

In control Runx3^fl/fl^ mice, we found that DAC combined with PD-1 antibody promoted the infiltration of T cells and the secretory ability of TILs (IFN, TNF-α). The proliferation ability (Ki67) and killing ability were significantly increased (GranB, Perforin, etc.), and this promoting effect was significantly improved after using DAC. For Runx3-knockout mice, the secretion, proliferation and killing ability of T cells were inhibited, suggesting that Runx3 may be the key mediator of the epigenetic sensitization function of DAC (Fig. 5c, e).

### Runx3 predicts the anti-PD-1 immunotherapy responses in a spectrum of tumor types

We deemed that it would be interesting to investigate whether Runx3 levels can predict the immune response to anti-PD-1. Immunotherapy has transformed the treatment landscape for a variety of tumors and has demonstrated durable response rates in some refractory tumors, yet unresponsiveness and severe immune-related side effects have been reported in some treated patients. Therefore, biomarkers are urgently needed to screen people who can benefit from immunotherapy.

**Fig. 6:**
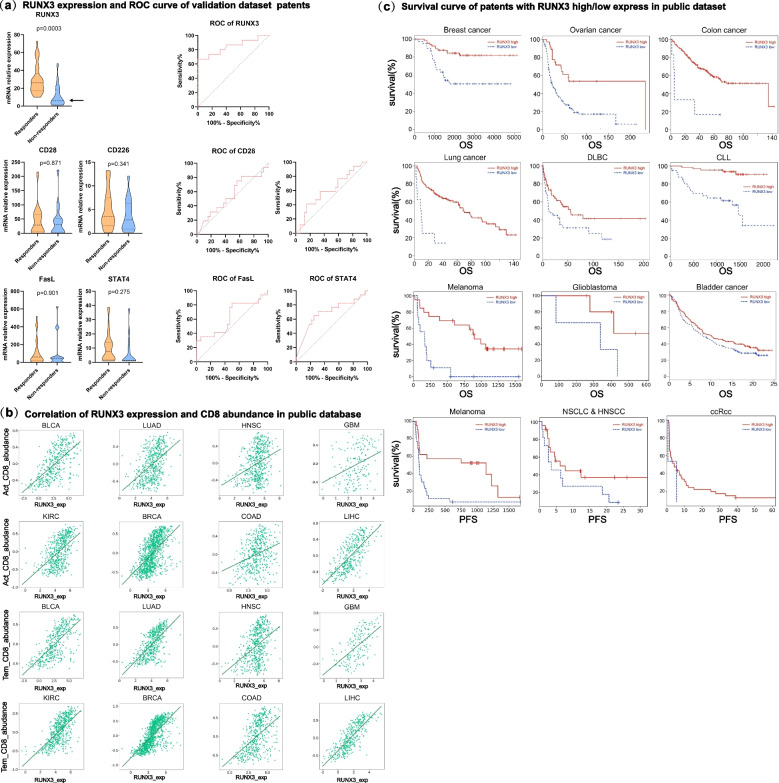
Runx3 is a key molecular marker of the clinical response to immunotherapy. **a** Violin diagram showing the expression levels of Runx3, CD28, CD226, FasL and STAT4 in T cells in responders and nonresponders. The figure shows the median, upper quartile and lower quartile. Two-tailed unpaired t tests. **b** Correlation analysis between effector T cells, memory T cells and Runx3. The x-axis represents Runx3 expression in T cells, and the y-axis represents the abundance of memory T cells or effector T cells. **c**. Kaplan‒Meier survival curves between high and low expression of Runx3 in T cells and prognoses in different cancer types

From a previous clinical study, our results showed that there was a strong correlation between the Runx3 expression and the clinical response (Fig. 6a). The receiver operating characteristic (ROC) curve, which is a useful graphical tool for assessing the predictive performance of a biomarker (Fig. 6a), indicated that a biomarker panel distinguished two groups: the responsive and non-responsive groups. ROC curves are universally used standards to evaluate biomarkers. We drew a ROC curve of Runx3 with the clinical response and found that the Runx3 level could crucially predict the clinical response. We show in Fig. 6b that Runx3 levels were associated with effector T-cell levels and memory T levels, not overall CD8+ T cell levels. Therefore, Runx3 contributes to the clinical ICB response by influencing the functional differentiation but not the overall level of CD8+ T cells. Noteworthy, when we treated patients with DAC, although the Runx3 level increased, the overall level of CD8+ T cells did not change, which indicates that this effect is not caused by changes in the number of T cells (Figure S5). The above data showed that number of functional T cells, but not overall level of T cells plays important role for the clinical response.

In addition to our clinical cohort, we explored the TISIDB database and analyzed the correlations of Runx3 levels and clinical prognoses of anti-PD-1 therapy. We found that Runx3 levels correlated well with clinical prognosis and survival rate. High expression of Runx3 was related to a favorable prognosis of anti-PD-1 regimen in patients with colorectal cancer, breast cancer and lymphatic cancer. This suggests that Runx3 is an important regulatory factor in anti-PD-1 immunotherapy and a potential biomarker for prognosis prediction. Taken together, these data show that Runx3 is not only a key mediator of DAC and CD8+ T cell function but also a potential biomarker for the clinical immune response.

## Discussion

PD-1 (programmed cell death protein 1) antibody therapy is a type of immunotherapy and an important part of immune checkpoint inhibitor therapy [6–13]. PD-1, an important immunosuppressive molecule, belongs to the immunoglobulin superfamily and is a membrane protein of 288 amino acid residues [7, 13]. Immunomodulation targeting PD-1 is of great significance in antitumor, anti-infection, and anti-autoimmune diseases and organ transplantation survival. Its ligand PD-L1 can also be used as a target, and the corresponding antibody can also play the same role. The binding of PD-1 and PD-L1 initiates the programmed death of T cells, enabling tumor cells to undergo immune escape. The PD-1 antibody can block the binding of PD-1 and PD-L1, thereby activating immune cells and killing tumors [16–19]. There are currently 6 PD-1 inhibitors on the market or approved by the FDA, which has greatly changed the status quo of tumor treatment. Although anti-PD-1 therapy has excellent therapeutic effects in many cases, it often faces the problem of a low clinical response rate.

For example, in melanoma, the response rate of patients with CTLA-4 or PD-1 blockade is only 20%-40% [14]. The ability and exhaustion are different. In addition, in breast cancer, the expression of PD-L1 is unstable; there is a lack of immune cells in the tumor microenvironment, and a large number of suppressor T cells infiltrate the tumor. The presence of immunosuppressive signals and the exhaustion of T cells will affect the response rate of PD-1 antibodies [16]. The clinical response rate of PD-1 antibody therapy in relapsed and refractory Hodgkin lymphoma is only 15–31% [17, 18]. How to improve the clinical response rate of PD-1 antibody treatment in refractory Hodgkin lymphoma is an important question. The scientific solution to this important clinical problem involves breakthroughs in related mechanism research and molecular marker research [31–33]. Research on PD-1 inhibitors is very popular, and new inhibitors are continuously emerging, but there has been no substantial progress in research on the mechanism of the low clinical response rate.

Recent studies have suggested that epigenetics is involved in the functional development of multiple subtypes of T cells [23–26]. Decitabine, the strongest known DNA-demethylating drug, plays an active role in the treatment of tumors [27–29]. In our previous study, low dose of the DNA-demethylating drug decitabine was used to enhance the antitumor efficacy of PD-1 inhibitors. This regimen significantly improved the complete remission rate of relapsed and refractory Hodgkin lymphoma, causing it to exceed 70%, which was at least 2 times higher than that of the single-drug group [30]. Subsequent studies have shown that the antitumor ability of decitabine-treated CAR-T cells is enhanced by 30–100 times, that the expansion ability is increased by more than 10 times, and that the cells can accumulate in large numbers at tumor sites and exist in the body for a long time. CAR-T cells undergo significant epigenetic reprogramming that influences the effector function of the cells. This suggests that low-dose decitabine-sensitized immunotherapy may be related to the epigenetic regulation of genes related to important functions of T cells. In-depth research on epigenetic alterations in T cells may be a key entry point to unlock the mechanisms of immunotherapy response rates.

Through dynamic analyses of different periods, we found that this demethylation occurred in the c1d6 period and could be maintained for at least one anti-PD-1 treatment cycle. The half-life of DAC is only 4 h, but the actual observed clinical effect of DAC lasts as long as 28 days. Given the results of our data analysis, we speculate that DAC in fact participates in the epigenetic reprogramming process of CD8 + T cells, thus reversing the epigenetic state of T cells. We also analyzed the functional enrichment of the differential methylation sites and found that the change in methylation pattern was enriched in important signaling pathways such as immune activation and T-cell activation, consistent with the improvement in immune function found in the clinic.

Decitabine exerts broad-spectrum effects. Ghoneim et al. obtained important findings that blocking de novo DNA methylation in activated CD8+ T cells allowed retention of effector functions despite chronic stimulation and preserved the ability of these T cells to respond effectively to ICB. However, Ghoneim et al. used DNMT3A-KO mice to study the underlying mechanism, which might have caused genome-wide methylation changes. Instead, we identified an important role of the specific gene Runx3 and generated Runx3-KO mice to study the function of Runx3 in CD8+T cells.

To study the mechanism in depth and elucidate the specific pathways, we needed to find the key molecules most closely related to the change in T-cell function upon broad-spectrum regulation. Because DNA methylation reprogramming can regulate gene expression, we conducted a multiomics analysis on CD8+ T cells from patients. Our results further confirmed that this methylation reprogramming is reflected in the expression profile. The differentially expressed genes were mainly enriched in immune-related pathways and were highly consistent with the methylation findings. Through screening, we found that Runx3 may be a key molecule regulated by DAC that is related to the prognosis of patients. Notably, studies have confirmed that Runx3 may play a regulatory role in the development of CD8 + T cells. [32–34]

Therefore, we analyzed the relationship between Runx3 and the prognosis of patients through a database. We found that abnormal expression of Runx3 was significantly correlated with prognosis in colorectal cancer, breast cancer and lymphoma.

Since Runx3 is also an important tumor suppressor gene, to demonstrate that the enhanced immune response we observed was due to T-cell-specific Runx3 function rather than an indirect result of tumor cells, we constructed mice with conditional knockout of Runx3 in T cells. In other words, to confirm the T-cell-specific function of Runx3, we explored the important role of the Runx3 gene through a conditional knockout mouse model. The results showed that knockout reduced the number of CD8+ T cells and significantly decreased the proportions of Th cells and CD3+ T cells related to adaptive immunity in the peripheral blood of mice. In the spleen, the proportions of innate immune cells such as DCs and APCs decreased significantly, as did the proportions of Th cells and CD3+ T cells involved in adaptive immunity. In the lymph gland, the proportions of innate immune cells such as DCs and APCs decreased significantly, as did the proportions of Th cells and CD3+ T cells involved in adaptive immunity. Comprehensive analysis of the secondary immune system suggested that Runx3 increased the proportion of CD8+ cells, reduced the content of effector cells and interfered with the balance of immune cell subtypes. In addition, we found that Runx3 knockout attenuated the function of DAC in promoting T-cell infiltration and caused the response difference and survival level between the P group and PD group to become comparable. We also drew survival curves based on the Runx3 levels in T cells, not in tumor cells. Taken together, the above findings minimize the interference of Runx3 from tumor cells. Even if Runx3 in tumor cells contributes somehow to tumor progression, it might work through pathways other than the immune system.

One of the three major clinical problems of current immunotherapy is the lack of biomarkers. Immunotherapy has transformed the treatment landscape for a variety of tumors and has demonstrated durable response rates in some refractory tumors, yet unresponsiveness and severe immune-related side effects have been reported in some treated patients. Therefore, immunotherapeutic markers are urgently needed to help screen people who can benefit from immunotherapy not only to avoid unnecessary costs for treatment nonresponders but also to avoid hyperprogression and potentially severe toxicity.

Some studies have shown that PD-L1 expression in tumors and the tumor mutational burden (TMB) are related to the efficacy of ICB. However, these conclusions are only applicable to some tumor types. The clinical application of these PD-L1 and TMB relationships is also limited due to the need to detect tumor specimens. If biomarkers that predict the efficacy of ICB can be found in blood, it will be of great benefit to clinical decision-making. At present, research on predictive markers of immunotherapy mainly focuses on positive predictors of efficacy, such as programmed death-ligand 1 (PD-L1), tumor mutational burden (TMB), microsatellite high instability (MSI-H) and mismatch repair deficiency (dMMR). Biomarkers for negative efficacy prediction mainly include mutations in specific genes, immunosuppressive molecules, or immunosuppressive cells. Among the biomarkers, PD-L1 and TMB are the most widely used clinically. We screened a clinical cohort and found that Runx3, a key molecule, has specific methylation changes and can predict the clinical response rate of decitabine combined with anti-PD-1 therapy. Our expanded clinical cohort combined with data from public databases showed that Runx3 also had good predictive efficacy for anti-PD-1 clinical response rates and survival curves. Changes in Runx3 can be detected in blood in the clinic. The mechanism is clear, and the detection method is convenient, so Runx3 can potentially be used as a rare biomarker for immunotherapy.

## Conclusion

We demonstrate that the DNA methylation of Runx3 plays a critical role in CD8 + T-cell infiltration and differentiation during decitabine-primed PD-1-ab immunotherapy, which provides a supporting mechanism for the essential role of epiregulation in immunotherapy.

## Supplementary Information

**Additional file 1: Figure S1.** Large scale demethylation is initiated by DAC. **Figure S2.** Dynamic expression changes of important immune related genes. **Figure S3.** Tsne analysis of T cells in peripheral blood and spleen after Runx3 conditional knockout in CD8+T cell. **Figure S4.** Tsne analysis of T cells in peripheral blood and spleen after PD-1 and DAC+PD-1 treatment in Runx3^flfl^ mice. **Figure S5.** CD8+ level did not changed when treated with PD-1 or DAC/PD-1. **Table 1.** Patient charcateristics. **Table 2.** Primer sequences for mRNA quantification. **Table 3.** Antibodies for flow cytometry. **Table 4.** Antibodies for Mass cytometry.

## Data Availability

Disclosures provided by the authors and data availability statement (if applicable) are available with this article at https://ngdc.cncb.ac.cn/search/?dbId=hra&q=HRA003337, (Accession number: HRA003337).

## Abbreviations

CTL: Cytotoxic t lymphocyte
DAC: Decitabine
FACS: Fluorescence activated cell sorting
FBS: Fetal bovine serum
MCHL: Mixed-cell Hodgkin’s lymphoma
NSHL: Nodular sclerosis Hodgkin’s lymphoma
PBMC: Peripheral blood mononuclear cell
PBS: Phosphate buffer saline
PD-1: Programmed death receptor 1
PD-L1: Programmed death receptor 1 ligand
Tils: Tumor infiltrating lymphocytes
ORR: Objective response rate
CAR-T: Chimeric Antigen Receptor T-Cell Immunotherapy
CR: Complete response
PR: Partial response
SD: Non-responders (patients with stable disease
PD: Progressive disease
OS: Overall survival
PFS: Progression free survival
DLBC: Diffuse large B cell lymphoma
CLL: Chronic lymphocytic leukemia
FACS: Flow cytometry analysis
CyTOF: Mass Cytometry
t-SNE: T-Distributed Stochastic Neighbor Embedding
DMSs: Differential DNA methylation sites
DEGs: Differentially expressed genes
TMB: Tumor mutational burden
MSI-H: Microsatellite high instability
dMMR: Mismatch repair deficiency
