# Supplementary material for: Epigenetic reprogramming of Runx3 reinforces CD8 + T-cell function and improves the clinical response to immunotherapy
Source: Mol Cancer. 2023 May 16;22:84. doi: 10.1186/s12943-023-01768-0 (PMC10186650; doi:10.1186/s12943-023-01768-0)
Supplement: Supplementary file 1 — Additional file 1: Figure S1. Large scale demethylation is initiated by DAC. Figure S2. Dynamic expression changes of important immune related genes. Figure S3. Tsne analysis of T cells in peripheral blood and spleen after Runx3 conditional knockout in CD8+T cell. Figure S4. Tsne analysis of T cells in peripheral blood and spleen after PD-1 and DAC+PD-1 treatment in Runx3flfl mice. Figure S5. CD8+ level did not changed when treated with PD-1 or DAC/PD-1. Table 1. Patient charcateristics. Table 2. Primer sequences for mRNA quantification. Table 3. Antibodies for flow cytometry. Table 4. Antibodies for Mass cytometry. [file 12943_2023_1768_MOESM1_ESM.docx]

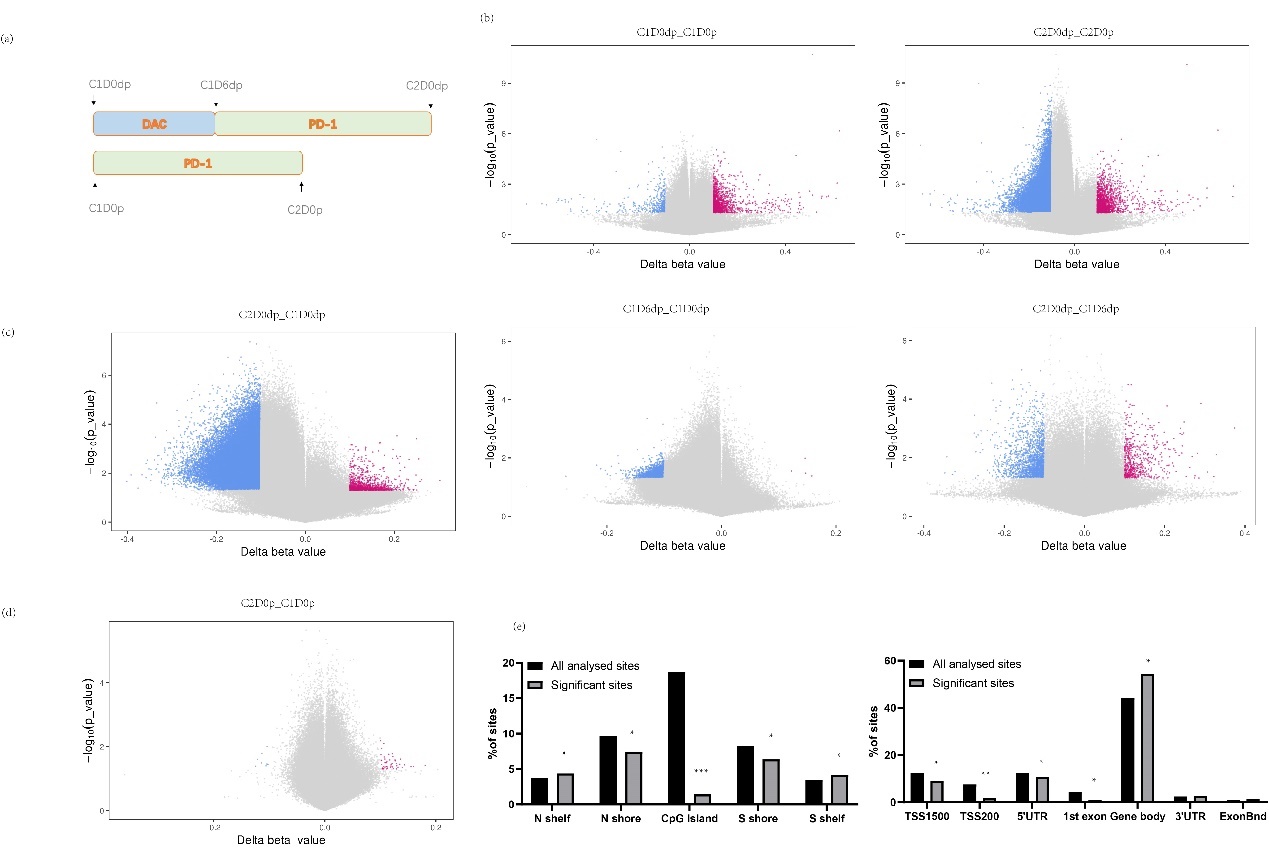


**Figure S1 Large scale demethylation is initiated by DAC**

a Basic mode of patient administration

b-d Analysis of genome-wide methylation difference of CD8 + T cells between different period. screened according to |Diff beta value ≤0.1|，P < 0.05. Blue represents low methylation sites and red represents high methylation sites

e Distribution of differential genes at the whole genome level，left panel：analysis by genomic region，right panel：analysis according to genomic functional domain(two-tailed unpaired t tests，*P <0.05，*,*P <0.01，***P <0.001)


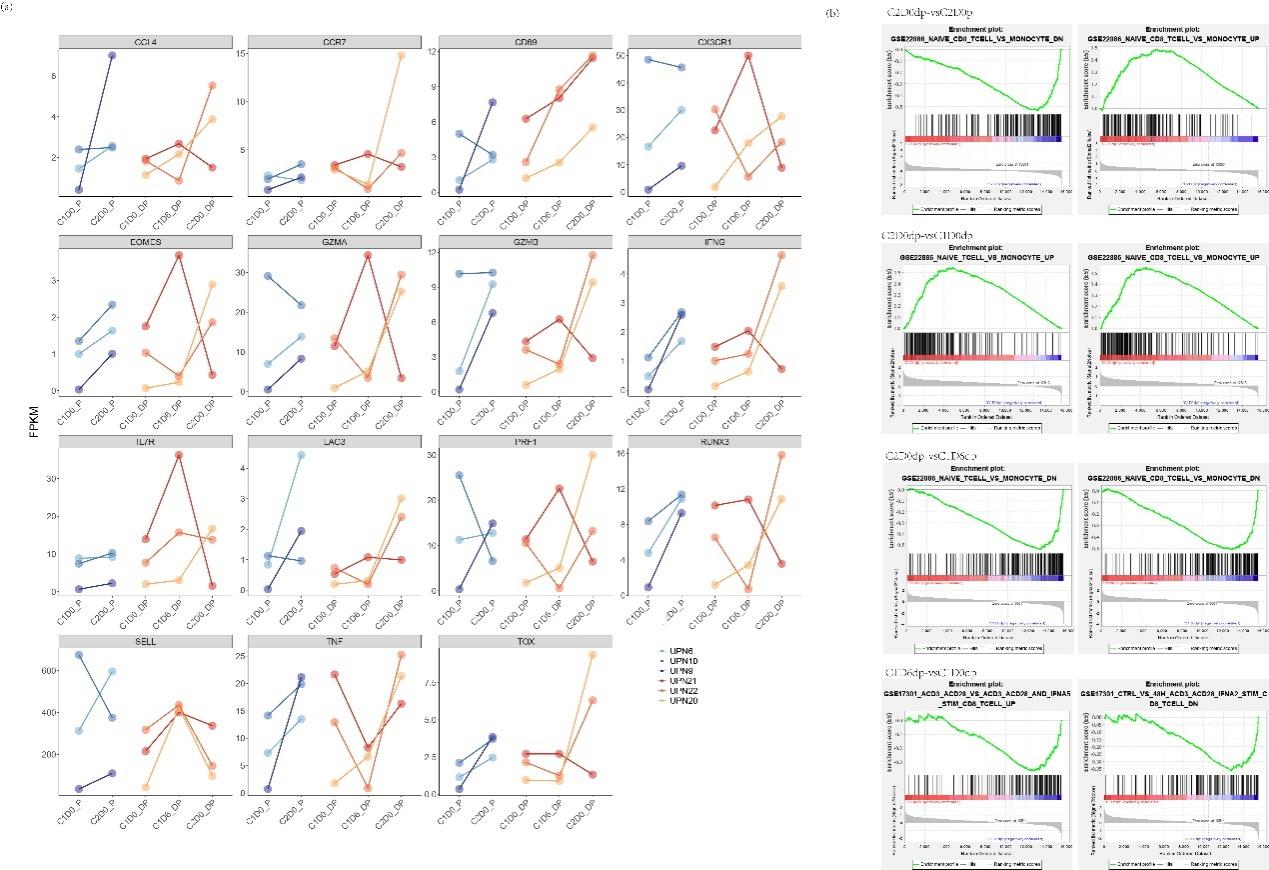


**Figure S2 Dynamic expression changes of important immune related genes**

a The expression levels of important immune related genes in different periods were analyzed by line diagram，The x axis represents the period and the y axis represents the fpkm value.

b tSEA analysis of DEGs in each period, The input data are DEGs of each period.


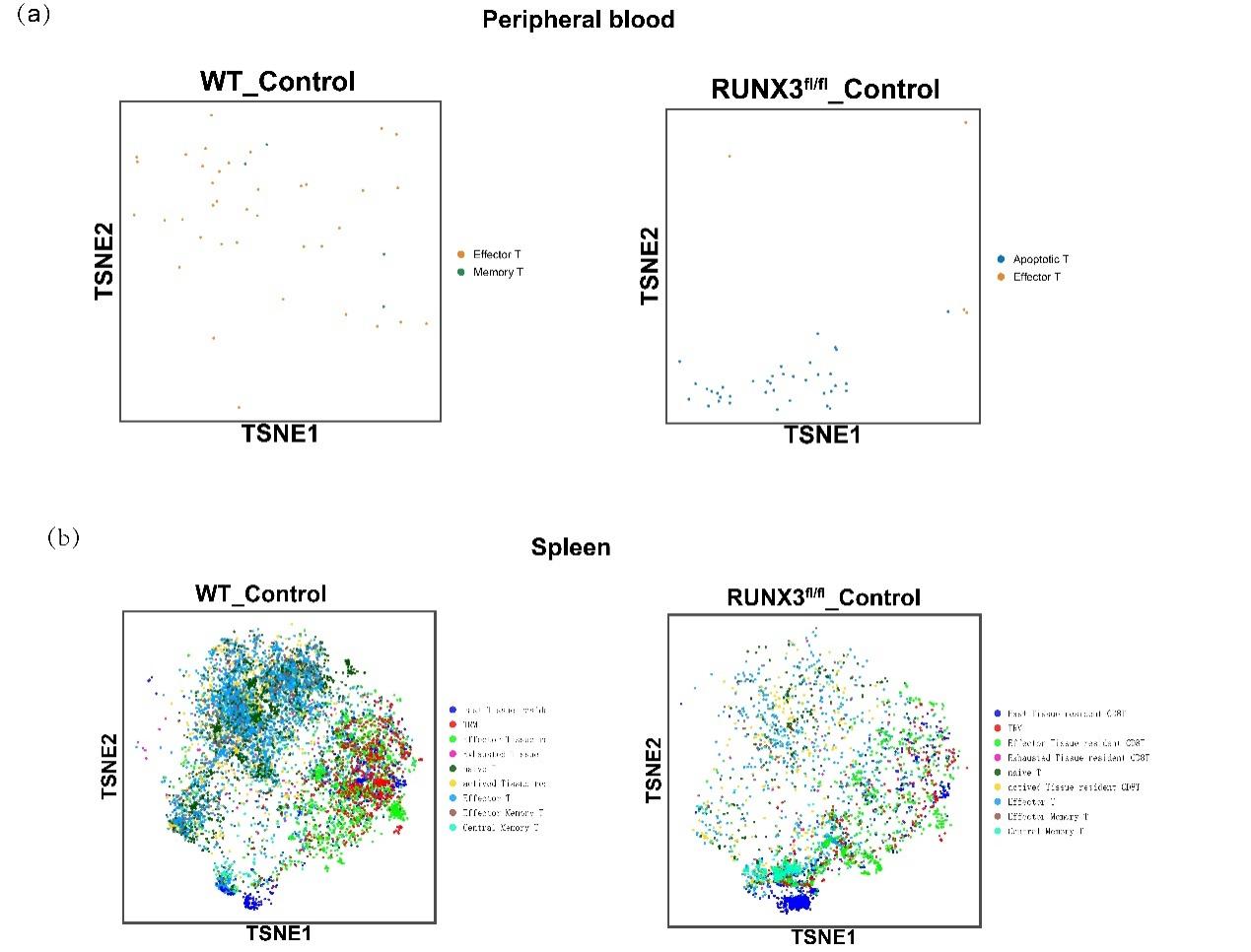


**Figure S3 Tsne analysis of T cells in peripheral blood and spleen after RUNX3 conditional knockout in CD8+T cell**

a Tsne analysis of T cells in peripheral blood, b Tsne analysis of T cells in spleen.


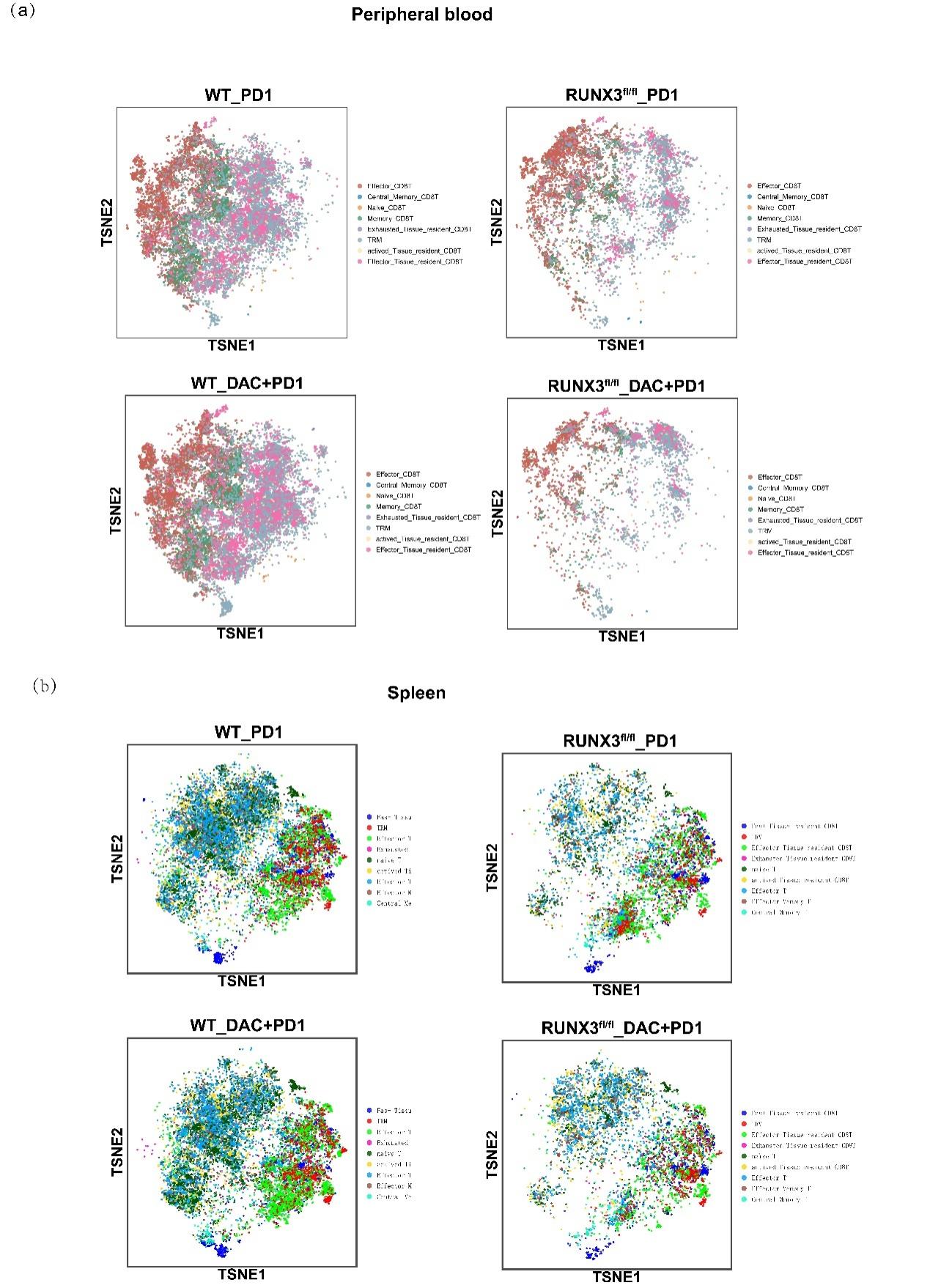


**Figure S4 Tsne analysis of T cells in peripheral blood and spleen after PD-1 and DAC+PD-1 treatment in RUNX3^flfl^ mice**

a Tsne analysis of T cells in peripheral blood after PD-1 and DAC+PD-1 treatment in RUNX3^flfl^ mice

b Tsne analysis of T cells in spleen after PD-1 and DAC+PD-1 treatment in RUNX3^flfl^ mice


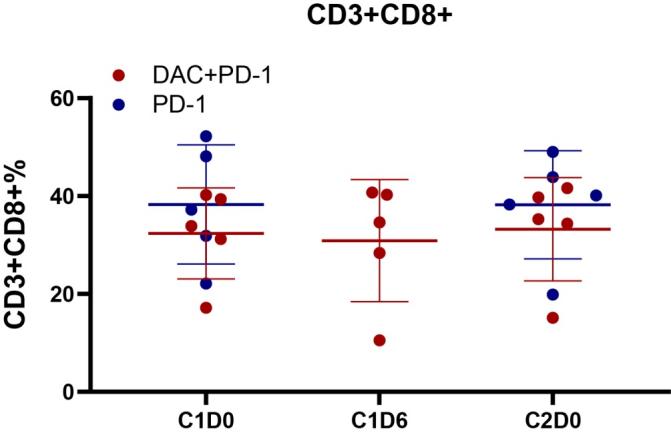


**Figure S5 CD8+ level did not changed when treated with PD-1 or DAC/PD-1.**

Y axis : CD3+CD8+ level (%) ; X axis: sampes of patients treated with PD-1 or DAC/PD-1

Triplicate samples were applies for each experiment and the median was shown as horizontal line (p>0.05)

## Table 1 Patient charcateristics

| **Patient No.** | **Age** | **Sex** | **Stage** | **Histology** | **Target lesion** | **Prior anti-PD-1 monotherapy** | **Cohort** |
| --- | --- | --- | --- | --- | --- | --- | --- |
| 6 | 26 | M | II | NSHL | Lymphoma nodes, lung, bone | No | Cohort 1 (Monotherapy) |
| 7 | 20 | M | II | NSHK | Lymphoma nodes | No | Cohort 1 (Monotherapy) |
| 8 | 24 | M | IV | NSHL | Lymphoma nodes, bone | No | Cohort 1 (Monotherapy) |
| 9 | 44 | M | IV | NSHL | Lymphoma nodes, bone, liver, spleen | No | Cohort 1 (Monotherapy) |
| 10 | 34 | F | IV | MCHL | Lymphoma nodes, bone, liver, spleen, lung | No | Cohort 1 (Monotherapy) |
| 21 | 36 | M | II | NSHL | Lymphoma nodes | No | Cohort 1 (Combination therapy) |
| 22 | 33 | M | III | MCHL | Lymphoma nodes | No | Cohort 1 (Combination therapy) |
| 25 | 18 | M | IV | NSHL | Lymphoma nodes, lung | No | Cohort 1 (Combination therapy) |
| 19 | 29 | F | II | NSHL | Lymphoma nodes | Yes | Cohort 2 (Combination therapy) |
| 20 | 27 | F | III | NSHL | Lymphoma nodes | Yes | Cohort 2 (Combination therapy) |

## Table 2. Primer sequences for mRNA quantification

| **Primer** | **Sequence (5′ to 3′)** |
| --- | --- |
| hRUNX3-F | GCAGGCAATGACGAGAACTA |
| hRUNX3-R | CAGTGATGGTCAGGGTGAAA |
| hSTAT4-F | GTATGCCAACCCACCCTCAG |
| hSTAT4-R | CACCATGTGACAGCCCTCAT |
| hCD28-F | TCCCTTCAATTCAAGTAACAGGAAA |
| hCD28-R | CCCGGAACTCCCTTGAGAAG |
| hCD226-F | GGGCATCTTAACACAGGTGGA |
| hCD226-R | ACAGCTGCCTCAAAACTATCTGA |
| hFASL-F | CTTGGTAGGATTGGGCCTGG |
| hFASL-R | CTGGCTGGTAGACTCTCGGA |
| hβ-actin-F | AGCACGGCATCGTCACCAACTG |
| hβ-actin-R | GAGCTGGAAGCAGCCGTGGCC |

**Table 3. Antibodies for flow cytometry**

| Antibodies | Clone | Company |
| --- | --- | --- |
| PerCP anti-mouse CD3ε | 145-2C11 | BioLegend |
| APC anti-mouse CD8a | 53-6.7 | BioLegend |
| FITC anti-mouse CD45 | I3/2.3 | BioLegend |
| PE anti-mouse Ki-67 | 16A8 | BioLegend |
| FITC anti-mouse Ki-67 | 16A8 | BioLegend |
| PE anti-mouse CD279 | RMP1-30 | BioLegend |
| FITC anti-mouse IFN-γ | XMG1.2 | BioLegend |
| PE anti-mouse IFN-γ | XMG1.2 | BioLegend |
| FITC anti-mouse TNF-α | MP6-XT22 | BioLegend |
| PE anti-mouse TNF-α | MP6-XT22 | BioLegend |
| PE anti-human/mouse Granzyme B Recombinant | QA16A02 | BioLegend |
| FITC anti-human/mouse  Granzyme B Recombinant | QA16A02 | BioLegend |
| PE anti-mouse Perforin | S16009A | BioLegend |
| PE Mouse IgG1, κ Isotype Ctrl | MOPC-21 | BioLegend |
| PE Rat IgG2a, κ Isotype Ctrl | RTK2758 | BioLegend |
| PE Rat IgG1, κ Isotype Ctrl | RTK2071 | BioLegend |
| FITC Rat IgG1, κ Isotype Ctrl | RTK2071 | BioLegend |
| FITC Rat IgG2a, κ Isotype Ctrl | RTK2758 | BioLegend |
| FITC Mouse IgG1, κ Isotype Ctrl | MOPC-21 | BioLegend |
| PE Rat IgG2b, κ Isotype Ctrl | RTK4530 | BioLegend |

## Table 4.Antibodies for Mass cytometry

|  | Clone | Metal channel | **Company** |
| --- | --- | --- | --- |
| CD45 | 30-F11 | 89Y | Biolegend |
| CD44 | IM7 | 113Ln | Biolegend |
| CD3e | 145-2C11 | 115ln | Biolegend |
| Ki67 | SolA15 | 139La | eBioscience |
| CD28 | 37.51 | 141Pr | Biolegend |
| CBFb | Polyclonal | 142Nd | BosterBio |
| KLRG1 | 2F1 | 143Nd | eBioscience |
| TNFa | MP6-XT22 | 144Nd | Biolegend |
| CTLA4 | UC10-4B9 | 145Nd | Biolegend |
| Eomes | Dan11mag | 146Nd | Invitrogen |
| CCR5 | C34-3448 | 147Sm | BD |
| CCR7 | 4B12 | 148Nd | Biolegend |
| CD38 | 90 | 149Sm | Biolegend |
| CD127 | A7R34 | 150Nd | Biolegend |
| STAT4 | 513710 | 151Eu | R&D |
| CD11c | N418 | 152Sm | Biolegend |
| CD62L | MEL-14 | 153Eu | Biolegend |
| Tbet | 4B10 | 154Sm | Biolegend |
| LAG3 | C9B7W | 155Gd | Biolegend |
| CD39 | 5F2 | 156Gd | Biolegend |
| TIGIT | 2190A | 157Gd | R&D |
| FasL | MFL4 | 158Gd | Biolegend |
| F4/80 | C1:A3-1 | 159Tb | BioRAD |
| SPP1 | Polyclonal | 160Gd | Leinco |
| CD49a | HMa1 | 161Dy | Biolegend |
| CD103 | 2E7 | 162Dy | Biolegend |
| CD25 | 3C7 | 163Dy | Biolegend |
| RORgt | 600214 | 164Dy | R&D |
| Fas | SA367H8 | 165Ho | Biolegend |
| RUNX3 | 527327 | 166Er | R&D |
| ICOS | C398.4A | 167Er | BioLegend |
| Foxp3 | FJK-16s | 168Er | eBioscience |
| CD49d | R1-2 | 169Tm | BioLegend |
| CCR3 | J073E5 | 170Er | BioLegend |
| PD1 | 29F.1A12 | 171Yb | BioLegend |
| Perforin | S16009B | 172Yb | Biolegend |
| Granzyme B | GB11 | 173Yb | Biolegend |
| IFNg | XMG1.2 | 174Yb | Bio-Xcell |
| IL2 | JES6-5H4 | 175Lu | Biolegend |
| TIM3 | RMT 3-23 | 176Yb | Biolegend |
| CD4 | RM4-5 | 197Au | Biolegend |
| CD8 | 53-6.7 | 198Pt | Biolegend |
